# Impact on Caesarean section rates following injections of sterile water (ICARIS): a multicentre randomised controlled trial

**DOI:** 10.1186/1471-2393-13-105

**Published:** 2013-05-03

**Authors:** Nigel Lee, Lena B Mårtensson, Caroline Homer, Joan Webster, Kristen Gibbons, Helen Stapleton, Natalie Dos Santos, Michael Beckmann, Yu Gao, Sue Kildea

**Affiliations:** 1 Mater Medical Research Institute, Mater Health Services, Brisbane, Queensland, Australia; 2 School of Life Sciences, University of Skövde, Skövde, Sweden; 3 Faculty of Health, University of Technology Sydney, Sydney, New South Wales, Australia; 4 Centre for Clinical Nursing, Royal Brisbane and Women’s Hospital, Herston, Queensland, Australia; 5 Faculty of Health Sciences, Australian Catholic University, Brisbane, Queensland, Australia; 6 University Centre for Rural Health, University of Sydney, Lismore, New South Wales, Australia

**Keywords:** Sterile water injection, Caesarean section rates, Back pain, Labour, Midwifery

## Abstract

**Background:**

Sterile water injections have been used as an effective intervention for the management of back pain during labour. The objective of the current research is to determine if sterile water injections, as an intervention for back pain in labour, will reduce the intrapartum caesarean section rate.

**Methods/design:**

***Design:*** A double blind randomised placebo controlled trial

***Setting:*** Maternity hospitals in Australia

***Participants*:** 1866 women in labour, ≥18 years of age who have a singleton pregnancy with a fetus in a cephalic presentation at term (between 37 + 0 and 41 + 6 weeks gestation), who assess their back pain as equal to or greater than seven on a visual analogue scale when requesting analgesia and able to provide informed consent.

***Intervention:*** Participants will be randomised to receive either 0.1 to 0.3 millilitres of sterile water or a normal saline placebo via four intradermal injections into four anatomical points surrounding the Michaelis’ rhomboid over the sacral area. Two injections will be administered over the posterior superior iliac spine (PSIS) and the remaining two at two centimetres posterior, and one centimetre medial to the PSIS respectively.

***Main outcome measure:***Proportion of women who have a caesarean section in labour.

***Randomisation:*** Permuted blocks stratified by research site.

***Blinding (masking):***Double-blind trial in which participants, clinicians and research staff blinded to group assignment.

***Funding:***Funded by the National Health and Medical Research Council

***Trial registration:***Australian New Zealand Clinical Trials Registry (No ACTRN12611000221954).

**Discussion:**

Sterile water injections, which may have a positive effect on reducing the CS rate, have been shown to be a safe and simple analgesic suitable for most maternity settings. A procedure that could reduce intervention rates without adversely affecting safety for mother and baby would benefit Australian families and taxpayers and would reduce requirements for maternal operating theatre time. Results will have external validity, as the technique may be easily applied to maternity populations outside Australia. In summary, the results of this trial will contribute High level evidence on the impact of SWI on intrapartum CS rates and provide evidence of the analgesic effect of SWI on back pain.

## Background

**Figure 1:**
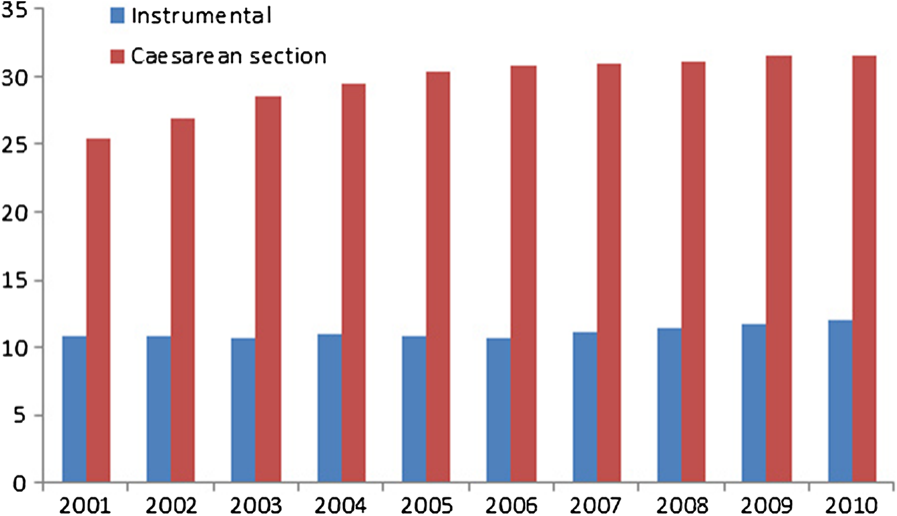
**Caesarean section and instrumental births, 2001 to 2010 **[1]**.**

Caesarean sections (CS) have risen annually in Australia for over 10 years reaching 31.6% of births in 2010 [1] (Figure 1), compared with the Organization for Economic Co-operation and Development average of 26% in 2009 [2]. A previous CS increases the likelihood of a repeat caesarean section; and is associated with major obstetric complications in subsequent births [3,4]; reduced fertility [5-8], and increased risk of ectopic pregnancy and spontaneous miscarriage [9]. Caesarean section has been associated with an increased likelihood of admission to neonatal intensive care nurseries [10-12] and an increased risk of neonatal mortality [13], and stillbirth in subsequent pregnancies [14,15].

**Figure 2:**
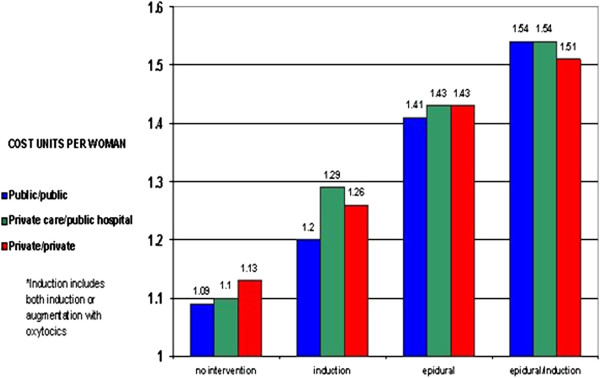
**Increase in costs as rates of intervention increase for low risk primiparous women in Australia (adapted with permission from **[16]**).**

The increasing CS rate imposes significant costs on health care systems with current Australian estimates placing the average cost for vaginal birth between $A3626-$A8907 compared with CS at $A8022-$A15229, depending on complications and insurance status [15]. Interventions in labour also add to the costs. For example, a cost analysis on the use of epidural anaesthesia in labour demonstrated an increase in the cost of vaginal birth by 30% and CS by 150% [16] (Figure 2). This cost calculation did not include the increased length of inpatient stay related to these interventions or other co-morbidities and complications that may occur.

### Lower back pain in labour – a significant problem

One in three women will experience continuous lower back pain in labour [17]. The occipito-posterior (OP) position of the fetus has been cited as a common contributing factor [18] although there is a paucity of evidence to support or refute this observation [19]. The OP position results in deflexion of the fetal head, which then presents a larger diameter as it enters and progresses through the maternal pelvis, hence contributing to obstructed labour and an increased risk of interventions including epidural anaesthesia and caesarean section [20]. Labour dystocia, characterised by very slow progress in labour together with significant and prolonged pain, further increases the risk of intervention, particularly epidural anaesthetic and CS [20]. Indeed, labour dystocia has been reported as the major reason for a CS during labour [21]. However, due to differences in Australian state and territory perinatal reporting, no data are available on the actual number performed for this reason. That said, a major Australian metropolitan hospital reported labour dystocia as the primary indication for 12.4% of all CSs performed in 2007 [22], with other research suggesting it was more common in women having their first baby (primiparous), accounting for nearly 80% of CSs performed during labour [23].

### Problems related to epidural for lower back pain in labour

Although epidural anaesthesia remains the most effective form of analgesia available to women in labour, substantial evidence describes a strong association between epidural use and persistent OP position and subsequent labour dystocia [20,24,25]. Epidural anaesthesia relaxes the pelvic floor muscles, disrupting the normal mechanisms of flexion and rotation of the fetal head that would otherwise facilitate a fetus in a malposition (OP) to self-correct [20,24,25]. There is some evidence to suggest that epidural use may be associated with an increase in instrumental birth and CS, particularly in nulliparous women [26]. Furthermore, epidural anaesthesia during labour is associated with co-morbidities arising from the need for urinary catheterisation to prevent urinary retention [27]. Urinary catheterisation is a primary source of urinary tract infection (UTI) with one study reporting an incidence of 30% amongst women who were catheterised during labour [28]; postpartum urinary retention and incontinence as a consequence of labour-related epidural use has also been reported [29-32].

### Sterile water injections as pain relief for lower back pain in labour

Sterile water injections (SWI) into the lower back can provide pain relief to women experiencing lower back pain during labour although there is still uncertainty about the effectiveness of this intervention on the improvement of clinical outcomes including decreased need for epidural anaesthesia or CS [33]. A sterile water injection, administered either intradermally or subcutaneously, causes osmotic and mechanical irritation resulting in a brief (15–30 second) but significant stinging sensation that is generally well tolerated by women. The onset of pain relief follows almost immediately in the majority of women and can last for up to two hours. The procedure can be repeated a number of times [34]. The method has been described as inexpensive to use and the injection technique is easy to learn [35].

### Review of previous trials of SWI in labour and effect on CS rates

A systematic review and meta-analysis [36] undertaken on SWI reported significant limitations. The meta-analysis included eight trials involving 828 women and found a CS rate of 4.6% in the sterile water injection groups and 9.9% in the control groups (RR 0.51, 95% CI: 0.30-0.87) [36]. Women in the control groups received normal saline placebo (six trials), acupuncture (one trial) and standard care defined as massage, water immersion and unrestricted mobilisation or transcutaneous electronic nerve stimulation (one trial). The authors reviewed the original publications of trial results and identified the following limitations:

•Only two reported a sample size calculation *a priori*[37,38]

•The method of allocation concealment was not described in two trials [39,40]

•It was unclear if the outcome assessment was blinded in two trials [39,41]

•Only one trial reported complete data on pain scores as many women gave birth prior to the final data collection point [37]

•Two trials did not report if additional modes of pain relief had been used following the SWI intervention [37,38]

•Only one of the investigators reported rates of cephalopelvic disproportion between groups [41].

The trials were all relatively small, were heterogeneous using different techniques for administering SWIs and assessing outcome measures, and contained a number of potential biases. With these limitations, it is impossible to attribute any relationship between lower CS rates in the sterile water group to the administration of SWI. Further, none of these trials had investigated the relationship between SWI and CS as the primary outcome; to our knowledge, no such study has been undertaken. The authors of the meta-analysis, noting the reduction in CS rates and the limitations in the current literature, recommended a large trial with CS as the primary outcome. The National Institute of Clinical Excellence (NICE) guidelines [33] also call for research into the effect of SWI on birth outcomes. With these issues in mind, we have designed the trial outlined in this protocol following the Consolidated Standards of Reporting Trials (CONSORT) guidelines [42].

### Gaps in the research on SWI as pain relief in labour

This will be the first clinical trial statistically powered to identify if the addition of SWI to standard maternity care is superior to standard care alone with CS rates as the primary outcome. Furthermore, there is limited information about analgesia, epidural anaesthesia use and birth outcomes following sterile water injections in labour. For example, it is not clear whether rates of instrumental vaginal deliveries are affected by the intervention. Combined results from the two trials [40,41] measuring this outcome show a trend towards an increased instrumental birth rate in the SWI group (OR 1.91; 95% CI 0.95-3.84). Finally, none of the trials have included a cost benefit analysis; we intend to undertake such an analysis.

## Methods/design

### Null hypothesis

H_0_: There will be no difference in the CS rate in women experiencing lower back pain in labour when comparing those who receive SWI with those who receive placebo.

H_1:_ There will be at least a 30 per cent relative difference in CS rates between the treatment groups.

### Aims

#### Primary aim

To determine if sterile water injections, as an intervention for back pain in labour, will reduce the in labour CS rate.

#### Secondary aims

To determine the effects of SWI for back pain in labour on:

•effectiveness for relieving back pain

•requirement for pain relief methods other than the intervention

•mode of birth and complications associated with birth (prior to hospital discharge)

•other maternal and infant outcomes in the immediate (six weeks) postnatal period

•cost of care for women and their babies during labour and birth, and the inpatient postnatal period from the perspective of the health system.

### Study design

A randomised, placebo controlled, double blind trial where participants receive either SWI or injections of a normal saline placebo. This trial will be conducted across maternity centres in Australia. The study protocol is registered on the Australian New Zealand Clinical Trials Registry (No ACTRN12611000221954). Six hospitals have confirmed involvement.

### Participants

Women in labour with lower back pain who satisfy the inclusion and exclusion criteria will be recruited from Australian maternity centres.

#### Inclusion criteria

Women who:

•In labour (spontaneous or induced, but not dependent on degree of cervical dilation)

•Are ≥ (equal to or greater than) 18 years of age

•Have a term singleton pregnancy (between 37 + 0 and 41 + 6 weeks gestation)

•Have a fetus in a cephalic (head down) presentation

•Experience back pain assessed by visual analogue scale VAS as ≥ 7 when women request analgesia for back pain

•Are able to provide informed consent.

#### Exclusion criteria

Women will be excluded if they fulfil any of the following criteria

•Multiple pregnancy

•Malpresentation (breech, transverse, shoulder)

•Previous CS

•Infection or inflammation at the injection sites or complications that could cause bleeding at injection site eg. Thrombocytopenia.

•Private insurance status

Private insurance status refers to women who utilise their health insurance to enable intrapartum care to be provided by the obstetrician of their choice. Only one site routinely offers women this model of care, whilst all sites offer obstetric care provided by publically a funded health system. Furthermore the use of private health insurance for obstetric care has also been associated with higher rates of CS than for those women receiving obstetric care through the public health system [43,44].

### Recruitment and consent process

This trial requires the recruitment and consent of women in labour. Pain associated with labour, together with the possible use of pharmaceutical analgesic agents including narcotics, may impact on a woman’s capacity to understand new information and make informed decisions, including whether to participate in research. By way of acknowledging the Australian National Statement on Ethical Conduct in Human Research [45] which recognises pregnant women as a potentially vulnerable group, consent processes in this study will reflect National Health and Medical Research Council (NHMRC) guidelines 4.1.1 to 4.1.10 [45]. That said, recent evidence suggests that women in labour are able to consent to participation in research, particularly when a staged approach is used [46]. This may be achieved by providing information well in advance of recruitment, for example during the antenatal period, which allows prospective participants time to consider it and discuss their potential involvement in the research with family members and significant others, including health care providers [46,47].

Recruitment will be undertaken at five metropolitan and regional maternity units in Queensland (QLD) and one metropolitan unit in New South Wales (NSW)). Hospital-based antenatal care providers will offer all eligible women a participant information and consent form (PICF) and discuss the purpose and design of the study during the antenatal period. As most participating hospitals, require women to return to the antenatal clinic in the third trimester of pregnancy (between 32 and 36 weeks), this provides an ideal opportunity to disseminate information, briefly discuss the study, and answer any immediate queries arising. At sites that do not employ this method of contact, the PICF will be provided at the time of booking into the hospital and distributed through the antenatal education classes. Consent will not be sought in the antenatal period. Contact details for the trial co-ordinators at each site are included on the PICF to enable women to seek more information if desired.

When women are in labour, they typically present to the Birth Suite of their respective hospital. Women will not be asked to consent to participation until such time as they have been fully assessed for eligibility against the inclusion and exclusion criteria. Although the majority of women will have received information regarding the trial during pregnancy, on admission to Birth Suite, staff will remind them of the trial and offer interested, and potentially eligible, women another copy of the PICF, and answer any outstanding questions.

Consent processes will be undertaken by the clinical trial co-ordinator at each site, or in their absence, a midwife who is not providing direct care to the woman. In our previous trial [48], women had no objections to consenting in labour, and when they were requesting analgesia for back pain. However as consent for clinical care differs from that required for research, if the person seeking consent considers that labour is too far advanced, or for any other reason staff consider women to be incapable of considering all available choices in clinical care, and of making an informed and voluntary consent, participation in the trial will not be sought.

Once the consent process has been completed, the woman will be randomised.

### Sample size

The meta-analysis of SWI use reported the overall rate of ‘in labour’ CS as 9.9% in the control group and 4.6% in the intervention (SWI) group [36] – i.e. a greater than 50% reduction in the CS rate in the SWI group. However, the review included trials where women receiving SWI were compared with women receiving either a placebo or other interventions (eg. acupuncture), and included trials that were conducted over two decades ago (1990 to 2008): a significant increase in CS rates (% in 1991 [49] to 31.5% in 2009 [50]) has been recorded in the intervening period.

More recently, at a metropolitan hospital in QLD, routine birth outcome data were collected on all women in labour who received SWI during 2010 (n = 427). The CS rate for women in the SWI group was 12.5%. The ‘in labour’ CS rate for women receiving standard care during the same period was 17.5%. This represents a difference of 30% which we suggest may conservatively reflect the contemporary clinical trend in CS rates. To demonstrate a similar reduction (17.5% to 12.5%), our study requires 839 women in each group (80% power, type I error 0.05). The attrition rate in our previous trial was 13% [48], however this was largely due to women giving birth prior to the measurement of the primary outcome (pain at 30 mins) and the use of nitrous oxide inhalation prior to randomisation. Neither of these are exclusion factors in this trial, therefore we expect attrition to be minimal and propose an attrition rate of 10%. The total sample size required is therefore 1886 women. As some sites have higher annual birth rates than other sites, the recruitment allocation anticipated for each site will be proportional.

### Definition of intervention, control and standard care

#### Intervention

**Figure 3:**
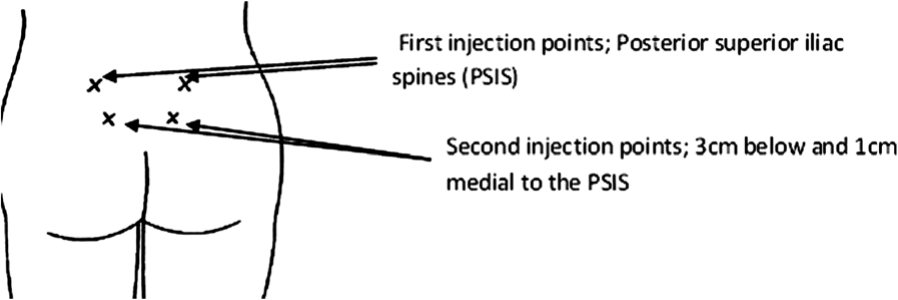
Injection sites for administration – control and intervention.

Participants in the intervention group will receive injections of 0.1–0.3 millilitres of sterile water for injection into four anatomical points surrounding the Michaelis rhomboid over the sacral area, two over the posterior superior iliac spines (PSIS) and the remaining two at two centimetres posterior, and one centimetre medial, to the PSIS respectively (Figure 3). This is a standard approach described in the majority of previous trials and has been demonstrated to be the most effective technique [34,48]. The actual volume injected, which may vary slightly depending on minor differences in tissue depth, is determined by a visual assessment of the resulting ‘bleb’ or blister. Minor discrepancies or changes to the anatomical position, depth or alignment of the four injections have not been shown to impact on any analgesic effect [51]. Injections will be given using 23 gauge needles or similar depending on actual equipment available at each site. One repeat injection will be provided for women who request them. Education sessions will be provided at all sites by the project manager, who has developed a dedicated ‘train the trainer’ course. All clinicians administering SWI will to be credentialed in this technique.

#### Control

Participants in the control group will receive 0.1 – 0.3 millilitres of normal saline 0.9% for injection into the same four anatomical points surrounding the Michaelis rhomboid as previously described. Repeat injections will also be provided as required (Figure 3).

#### Care during labour

All women will receive care throughout labour and birth as required according to routine Australian clinical practice; including access to the usual analgesic options. Preliminary planning using data from perinatal databases at each site reveals that non-pharmacological and pharmacological analgesic options are similar; and have been incorporated into our definition of ‘standard care’. Non-pharmacological options include water immersion, aromatherapy, massage, and hot showers. Pharmacological options include Nitrous Oxide inhalation, narcotics (pethidine and morphine) and epidural or spinal anaesthesia. Intervention rates (e.g., epidural and CS) are similar across sites. At a number of current and planned research sites SWI is not available as standard care (demonstrating the importance of research in this area). Sterile water injections are available at site one, two and four, and women requesting SWI at these sites will not be entered into the trial.

### Randomisation and blinding

Blinded (plain label) ampoules of sterile water (intervention) and normal saline (placebo) for injection will be prepared by the Mater Health Service Pharmacy for all sites, and labelled according to a randomisation schedule prepared by the Mater Medical Research Institute. The ampoules will be delivered to each of the sites and stored in a locked container. The randomisation schedule will use permuted blocks and will be stratified by research site. In our previous trial about 10% of participants requested a repeat dose, to provide for this with minimal crossover between groups (e.g., women randomised to normal saline receiving sterile water as a repeat dose) each allocation packet will include two ampoules of the allocated treatment. Following consent, two midwives (other than the midwife assigned to provide the woman’s care in labour, or ‘primary midwife’) will obtain the next ampoule in numbered order and administer the injections. As this is a double blind trial, the administering midwives will be unaware of the arm to which the participant has been allocated. The ampoule number will be recorded in a research log and on the data collection forms. As the injection of normal saline does not result in the same degree of administration pain as sterile water, both interventions will be administered by midwives other than the primary midwife, who will be absent from the room throughout. This will prevent the primary midwife from observing the woman’s reaction to the administration which reduces the likelihood of assumptions being made about the arm of the trial into which the woman has been randomised.

## Outcome measures

### Primary outcome

•Proportion of women who have a CS in labour

### Secondary outcomes

•Proportion of women undergoing induction and/or augmentation of labour

•Proportion of women receiving epidural anaesthesia

•Proportion of women having instrumental vaginal births

•Primary indication for CS

•Visual analogue scale of back pain prior to SWI

•Visual analogue scale of back pain following SWI (10, 45 minutes)

•Length of 1st stage and 2nd stage of labour

•Results of most recent vaginal assessment prior to randomisation

•Position of fetal occiput in relation to the maternal pelvis prior to randomisation and how this was determined

•Proportion of women breastfeeding at discharge

•Proportion of neonates admitted to the neonatal intensive care unit

•Cost of care from admission in labour until discharge of mother and baby from hospital from the perspective of the health system.

•Experience of satisfaction, relaxation, social support, negative side effects and memory of pain.

## Data management

### Data sources

Obstetric and neonatal outcome data will be obtained from either the onsite obstetric database where available or the Perinatal Data Collection unit in each state.

The VAS will be used to measure perceptions of pain. The VAS is an ungraded 100 mm long horizontal straight line with the endpoints “no pain” to the left (0) and “worst pain imaginable” to the right (100) [52]. To record a VAS, a participant marks a point on the line that matches the amount of pain experienced. The point is then quantified by measuring the distance in millimetres between “No Pain” and the point marked by the woman against a 0-10 cm gauge. The VAS has been shown to be sensitive to pain intensity and most individuals have no difficulties with its use [53].

Data for the cost effectiveness analysis (CEA) will be obtained from the perinatal database and will include data on interventions during labour and the postnatal period, length of stay, admission to neonatal nursery, mode of birth, and other analgesia requirements. In addition, data on staffing required during labour (number of midwives and time required from each midwife for care provided during labour) will be collected. Data on hospital costs will be obtained through the health service managers. These will include staffing, equipment and consumables for each woman and baby.

### Data entry and storage

Identified data collection forms will be kept in locked storage on each site prior to secure transfer to the Project Team. Upon receipt, data will be entered onto a specifically designed password protected database by designated research staff, all individual data will be de-identified through allocation of a unique study code. Data entry and storage, both paper and electronic will be managed and maintained as specified in the Human Research Ethics Committee (HREC) approvals for a period of not less than 15 years.

## Data analysis

### Statistical methods

All women who underwent randomisation will be analysed in their allocated treatment groups (ie. intention to treat). Relative risks with 95% confidence intervals for the primary outcome will be calculated. Secondary outcome measures of categorical data will be analysed with chi-squared tests and continuous data will be analysed with t-tests for normally distributed data and Mann–Whitney *U* test for parametric data. Regression will be used if necessary to adjust for any other confounding variables. All study outcomes will be analysed using a two-sided P value of < 0.05 to indicate statistical significance. The study is only powered to report the primary outcome and for secondary outcomes confidence intervals (CI) will be included as a measure of the effect size. Differences in pre and post VAS scores will be reported in the format recommended by the recent Cochrane Review [54].

### Economic analysis

The cost-consequences analysis will be undertaken from the perspective of the hospitals to determine if the sterile water injection is a cost-effective measure to reduce back pain in labour compared to the normal saline injection [55]. Direct costs will be measured for mother and baby from consent to participate in the study until discharge from hospital and mothers and babies re-admission to the same hospital within 28 days of births. Information about resource use and costs will be collected at each site. The study will conduct a bottom-up costing for the intervention and pain-relief methods at each site. The costs included in the study are: costs for the intervention (1 ampoule of sterile water or normal saline (10 ml), 2 x 1 ml syringes, 2x1 25 gauge needles, 2 alcohol prep swabs and the midwives’ time to administer the intervention, costs of care in labour, birth, postnatal and neonatal admission, and re-admission costs within 28 days of births. Costs will be allocated to the relevant resource items using appropriate values. For example, staff time will be costed using hourly award rates plus on-costs; and the costs of care in labour, birth and postnatal in hospitals will be costed using appropriate Diagnosis Related Groups.

### Study duration

Study enrolment will commence after HREC approval is obtained at each site. Enrolment is expected to continue for 18–24 months across all sites; recruitment rates have been conservatively estimated based on our previous trial. The study duration will be three years and the primary endpoint will be time of birth.

## Trial governance

To assist with trial governance Collaborative Trial Agreements (CTA) will be established between each of the trial sites and the sponsor (ACU). Site investigators that are signatories to the agreements will be required to provide reports on site activities at the quarterly Research Team meetings and liaise with the Project Manager on an ongoing basis. Case report data on each participant will be collected directly from state and/or hospital databases, de-identified and stored on a password protected database. The case report data will be managed by the Project Leader, or designated research team member and made available to Principal Investigator.

## Staff training

### Administration of SWI

Sterile water injections are currently in use at some sites. Each of the sites in which SWI is already utilised have developed protocols, clinical guidelines for the use of SWI and training and competency documents based on information provided previously by the members of the research team. Therefore there is already a standard approach to SWI at these sites. A number of the sites do not currently use SWI and will be provided with training materials by the researchers, forms for assessing competency is contained within the trial documentation for use by these sites. The training will consist of workshops provided by the researchers and access to an online education resource. This resource will be available to all staff at each of the sites and can be accessed via any internet connected computer. This process will ensure a consistent approach to the administration of the interventions. The procedure is more commonly undertaken by midwives, however particularly in regional sites, other clinicians for whom the administration of injections is a normal part of their scope of practice, may also receive training in SWI administration.

### Study processes

The researchers will hold regular in-service sessions at each site to provide staff with information about the study processes and procedures, specifically, participant recruitment, consent and data collection. The in-service sessions together with support from the site co-ordinators will ensure trial integrity across sites. As consent will be undertaken during labour, to ensure similar information is provided to all women during the consent process, a guideline script will be provided to staff for use when discussing the trial.

## Ethical aspects

Human Research Ethics Committee (HREC) approval for all current sites has been provided by the Royal Brisbane and Women’s Hospital HREC, according to the single site HREC approval for multicentre clinical trials guidelines. Research governance approval has been provided by the Research Governance Office at each site.

### Potential risks

No adverse events, allergic or systemic reactions to the procedure have been reported in the literature other than the brief stinging sensation immediately following administration [51].

### Data safety and monitoring

A multidisciplinary (statistician, clinical expert and research expert) Data and Safety Monitoring Committee (DSMC) will review all adverse events, which will also be reported by site staff to the participating institution’s Human Research Ethics Committee. In the case of complications or adverse events, participants will continue to receive routine care and have access to the research staff named in this protocol. The DSMC, which will be blinded to treatment allocation, will undertake interim analysis for safety and/or efficacy once 50% of the women have enrolled. The interim analysis will assess for any untoward outcomes and ensure sample size and power calculations are appropriate based on the observed difference between groups for the primary outcome.

### Interruption of the study

The CIs may terminate this study prematurely, either in its entirety or at individual study centres, for reasonable causes (e.g. unsatisfactory enrolment with respect to quantity or quality, inaccurate or incomplete data collection, falsification of records, failure to adhere to protocol). In such a case, a written notice of the intended termination will be sent to the site investigator. The site investigator may also terminate the study prematurely at his/her study centre for reasonable cause, after providing a written notice to the CIs at least 4 weeks prior to the intended date of termination.

## Discussion

In 2008 over 80,000 women in Australia underwent CS [56]. The rising CS rate, and associated, social, medical and economic impacts, has been recognised by governments and policy makers as a key priority area calls for future research to specifically target CS rates [15]. Indeed, the New South Wales Department of Health [57] has set a State-wide target for health services to reduce CS rates to 20% by 2015, through employing strategies such as non-pharmacological analgesia for women in labour. Sterile water injections, which may have a positive effect on reducing the CS rate, have been shown to be a safe and simple analgesic suitable for most maternity settings. However, results of a recent cross sectional study of SWI use amongst Australian midwives indicated that it was not routinely available as an analgesic choice for labouring women in most maternity units [58], lack of robust evidence in this area may help to explain why the technique is not more widely used. A procedure that could reduce intervention rates without adversely affecting safety for mother and baby would benefit Australian families and taxpayers and would reduce requirements for maternal operating theatre time. Results will have external validity, as the technique may be easily applied to maternity populations outside Australia. In summary, the results of this trial will contribute:

1. High level evidence on the impact of SWI on intrapartum CS rates

2. High level evidence of the analgesic effect of SWI on back pain.

## Study administration

Personnel

### Principal investigators

Sue Kildea, Lena Martensson; Joan Webster, Caroline Homer, Helen Stapleton, Michael Beckmann, Yu Gao, Kristen Gibbons.

### Associate investigators

Maree Reynolds, Ian Jones, Bob Baade, Cathy Styles, Louis McPherson, Jennifer Kelly, Patricia Smith, Beth Hartley, Vicki Carson, Audrey Simpson, Donna Hartz.

### Project team

Sue Kildea, Nigel Lee, Helen Stapleton, Natalie Dos Santos, Sara Menke.

### Project leader

Nigel Lee

### Project co-ordinator

Natalie Dos Santos

## Abbreviations

ICARIS: Impact on caesarean section rates following injections of sterile water; PSIS: Posterior superior iliac spine; CS: Caesarean section; OP: Occipto posterior; UTI: Urinary tract infection; SWI: Sterile water injection; NICE: National institute of clinical excellence; CONSORT: Consolidated standards of reporting trials; VAS: Visual analogue scale; NHMRC: National health and medical research council; QLD: Queensland; NSW: New South Wales; SA: South Australia; PICF: Participant Information and consent form; CI: Confidence intervals; HREC: Human research ethics committee; DSMC: Data and safety monitoring committee; CALD: Culturally and linguistically diverse; CEA: Cost effectiveness analysis; CTA: Collaborative trial agreements; ACU: Australian Catholic University.

## Competing interests

The authors declare that they have no competing interests.

## Authors’ contributions

SK is chief investigator and has overall responsibility of the trial; NL, SK originally conceived the study; SK, NL, JW CH LM designed the study; SK, NL wrote the initial grant application; SK, NL JW CH drafted the initial study protocol; LM NL drafted the questionnaires; SK, NL, NDS, SM reviewed the questionnaires; SK, NL, LM, conceived the intervention; NL NDS Implemented and co-ordinated the trial; NL KG SM Developed data collection processes; NDS NL Data collection and management responsibility; NL KG SK LM Will undertake data analysis; NL NDS SK Drafted the trial protocol manuscript: All authors read and approved the final manuscript.

## Pre-publication history

The pre-publication history for this paper can be accessed here:

http://www.biomedcentral.com/1471-2393/13/105/prepub
